# Streetlights positively affect the presence of an invasive grass species

**DOI:** 10.1002/ece3.7835

**Published:** 2021-07-10

**Authors:** Shannon M. Murphy, Dhaval K. Vyas, Jennifer L. Hoffman, Clara S. Jenck, Brooke A. Washburn, Kelsie E. Hunnicutt, Angie Davidson, Jasmine M. Andersen, Rachel K. Bennet, Amarin Gifford, Mia Herrera, Braelynn Lawler, Sophie Lorman, Vanessa Peacock, Lyndi Walker, Esme Watkins, Lakyn Wilkinson, Zariah Williams, Robin M. Tinghitella

**Affiliations:** ^1^ Department of Biological Sciences University of Denver Denver CO USA; ^2^ Department of Physics and Astronomy University of Denver Denver CO USA; ^3^ Fox Ridge Middle School Aurora CO USA; ^4^ Denver Language School Denver CO USA; ^5^ Assumption Catholic School Denver CO USA; ^6^ Girls Athletic Leadership School Denver CO USA; ^7^ Colorado Skies Academy Englewood CO USA; ^8^ Denver School of the Arts Denver CO USA; ^9^ John Wesley Powell Middle School Littleton CO USA; ^10^ Laredo Middle School Aurora CO USA

**Keywords:** artificial lights, cheatgrass, light pollution, plant invasion

## Abstract

Anthropogenic disturbances associated with urban ecosystems can create favorable conditions for populations of some invasive plant species. Light pollution is one of these disturbances, but how it affects the growth and establishment of invasive plant populations is unknown. Cheatgrass (*Bromus tectorum*) is a problematic invasive species where it has displaced native grassland communities in the United States, but to our knowledge, there have been no studies of the ecological factors that affect cheatgrass presence in urban ecosystems. We conducted field surveys in urban alleys in Denver, Colorado, to compare the presence of cheatgrass at sites with and without artificial light at night (hereafter artificial light) from streetlights. These streetlights are mounted on utility poles, which cause ground disturbance when installed in alleys; we were able to test the independent effect of poles on cheatgrass establishment because not all poles have streetlights on them. We found that cheatgrass was positively associated with the presence of streetlights and to a lesser extent poles. In addition to cheatgrass, we also found that other plants were positively associated with the presence of both poles and streetlights. Our results suggest that artificial light may benefit the occurrence of cheatgrass and other plant species in urban settings. While invasive populations of cheatgrass in wild habitats attract the most attention from managers, we suggest more consideration for this grass in urban environments where its growth and establishment benefit from anthropogenic changes.

## Graphical abstract

We have found that light pollution facilitates an invasive plant species, cheatgrass. Ours is the first study to document the effect of this global change driver, artificial light at night, on this important invasive plant species.

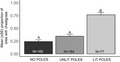

## 1 INTRODUCTION

Urban habitats contain unique environmental factors that affect plant fitness. Anthropogenic activity influences plant establishment and growth by altering air quality (Lombardozzi et al., 2012; Splash, 1997), water quality (Court Stevenson et al., 1993), heavy metal concentrations (Bucker‐Neto et al., 2017), nutrient subsidies (Murphy et al., 2012; Wimp et al., 2019), impermeable surfaces (Celestian & Martin, 2004), and artificial light (Bennie et al., 2016, 2018). However, native and non‐native plants may respond differently to environmental factors experienced within urban ecosystems. If non‐native invasive plants thrive under conditions in urban ecosystems that are detrimental to native species, then studying plant responses to anthropogenic disturbances in urban ecosystems enhances our understanding of invasion biology.

Artificial light is a ubiquitous disturbance in urban habitats and has been shown to affect plant phenology with plants leafing out and flowering earlier but senescing later (Bennie et al., 2016; ffrench‐Constant et al., 2016; Massetti, 2018; Skvareninova et al., 2017) and also plant physiology with increased leaf toughness, increased carbon: nitrogen ratios, and decreased photosynthetic efficiency among other effects (Grenis & Murphy, 2019; Meravi & Prajapati, 2018; Murphy et al., In Review; Xu et al., 2019). These changes in phenology and physiology likely alter plant community structure if plants vary in their responses to artificial light (Bennie et al., 2018). Artificial light also affects plant–plant interactions, such as competition for resources, and thereby alters competitive outcomes between species (Speißer et al., 2020). Artificial light can influence plant–animal interactions, which may cascade to affect how herbivores interact with their host plants and thus alter plant community structure. For example, some herbivorous insects have been found to be negatively affected directly by artificial light and also indirectly via altered physiological responses of their host plants to artificial light at night (Grenis & Murphy, 2019). In particular, invasive plants, which are often more resilient to disturbance (Lozon & MacIsaac, 1997), may gain competitive advantages from the extended photoperiods created by artificial light. While many studies demonstrate that artificial light affects plants, few studies focus specifically on how artificial light affects invasive plant species. Furthermore, urban ecosystems can be important to invasive species management because invasive species may thrive in these settings prior to entering natural areas (Alston & Richardson, 2006; McLean et al., 2017).

Cheatgrass (*Bromus tectorum*) is an annual grass that originates from Eurasia (Mack, 1981) and is invasive in the United States. Populations of this non‐native grass are predominant in the grassland ecosystems of the western United States where it was first recorded between 1889 and 1899 (Mack, 2011; Pawlak et al., 2015). Since its arrival, cheatgrass has displaced existing plant communities in over 20 million ha within the western US (Bradley et al., 2018; Duncan et al., 2004; Huttanus et al., 2011). High dispersal rates (e.g., via wind, human, or animal transport) and propagule numbers (a single plant can produce 25–5,000 seeds), as well as efficient acquisition of soil resources, all contribute to the success of cheatgrass (Bradford & Lauenroth, 2006; Mazzola et al., 2011; Zouhar, 2003). Cheatgrass also has significant societal and ecological impacts as it increases the intensity and frequency of wildfires, which can lead to economic damage within rangelands (Balch et al., 2013; Williamson et al., 2020). In grassland ecosystems, cheatgrass is often found in disturbed areas such as roadsides, railway tracks, and utility right of ways, all of which are common in urban habitats (e.g., Gelbard & Belnap, 2003; Mack et al., 2003; Mosely et al., 1999; Upadhyaya et al., 1986). However, most research on cheatgrass invasion has focused on natural grasslands (e.g., Mack, 2011; Owens et al., 2013; Pawlak et al., 2015), and to our knowledge, there are no studies of how urban environments facilitate the establishment of cheatgrass.

Previous work by some of the authors and our collective observations in the Denver metro area suggested that artificial light might be associated with the presence of invasive grasses, and in particular cheatgrass. To test the effects of artificial light on the growth and germination of native and invasive grass species, we previously grew six different grass species in a greenhouse either under ambient conditions or under streetlights; cheatgrass was the only species to grow significantly larger aboveground when grown under streetlights and indeed mean aboveground biomass was twice as great in the streetlight treatment compared with the ambient treatment (Murphy et al., In Review). However, greenhouse experiments do not always reflect patterns in nature (Forero et al., 2019) and whether streetlights facilitate cheatgrass growth outside in urban environments and not only in controlled greenhouse conditions has not yet been tested.

In this study, we aim to fill knowledge gaps regarding the urban ecology of cheatgrass and how it is affected by the disturbance of light pollution. We conducted field surveys to examine the effect of artificial light on plants, including cheatgrass, within an urban environment. Our research objective was to determine whether the presence of plants, and cheatgrass in particular, was affected by the presence of utility poles with and without streetlights installed upon them. Given the ground disturbance required to install poles, we expected to find plants and cheatgrass growing at more sites with poles than at sites without poles. Further, we expected that cheatgrass would be positively associated with the presence of streetlights.

## 2 METHODS AND MATERIALS

### 2.1 Study system

The city of Denver has alleys that run north–south behind residences and businesses located on the north–south streets. These alleys are 6 m wide and 185 m long and are endcapped at their north and south ends by streets that run east–west. We studied 54 alleys surrounding the University of Denver (DU), which is located in south Denver (Figures S1 and S2). We restricted our study to alleys with streetlights, which are mounted on wooden poles ~6–7 m above the ground; the poles are generally 30‐40m apart in the alleys and not all poles have streetlights (the non‐streetlight poles have electrical boxes on them). The plant community in these alleys consists mostly of weedy species, including a congener of cheatgrass (smooth brome, *Bromus inermis*) as well as dandelions (*Taraxacum officinale*), bindweed (*Convolvulus arvensis*), lambs quarters (*Chenopodium berlandieri*), purslane (*Portulaca oleracea*), and Canada thistle (*Cirsium arvense*). However, some homeowners have planted small gardens behind their houses and the plants behind these houses are more manicured.

### 2.2 Survey design and methods

We designed and began our study during the DU SciTech STEM camp, which took place 5–9 August 2019. DU SciTech is a week‐long camp for middle‐school girls and is designed to increase representation in STEM. Participation in extracurricular STEM activities can help middle‐school‐age students develop STEM‐related tools and skills and encourage them to view themselves as capable of succeeding in these fields (Broder et al., 2019) and a previous assessment of our camp showed particularly strong effects of participation on self‐reported scientific self‐efficacy (Broder et al., In Preparation). High STEM self‐efficacy is a strong predictor of career choice for girls (Larose et al., 2006) and positively influences the success of K‐12 racial and ethnic minority students (Museus et al., 2011). This research project was part of an inquiry‐based and community‐focused activity during the camp.

Together, we (campers and camp leaders) designed the survey to test whether cheatgrass was more likely to be found under poles with streetlights than under unlit poles or areas between poles. We surveyed the 54 alleys in our study between 7 and 28 August 2019; at this time of year, cheatgrass has produced seed but the seeds have not yet dropped, which makes it relatively easy to identify even by inexperienced botanists. We followed the same protocol in each alley. When we first entered an alley, we stopped at the first pole and recorded whether there was a streetlight, whether there were any plants present within a 5m radius of the pole (in the alley as the yards were blocked by fences and therefore not surveyed), and then if plants were present whether any of them were cheatgrass. Given that many in our group are not proficient plant taxonomists, we only noted the presence/absence of plants and cheatgrass in particular. We then went halfway between the first and second poles and recorded whether there were any plants and whether any of them were cheatgrass; sites between poles were as likely to have soil and adequate places for plants to grow (i.e., they were not entirely pavement) as pole sites (Figure S2). We then continued to record the presence of a streetlight, plants, and cheatgrass at each of the poles as well as the “between” sites (no pole sites in our analyses) in the alley until we reached the other end of the alley. Before we began our surveys, we confirmed that every one of the authors knew how to identify cheatgrass and understood our protocol. It is possible that some homeowners weeded their cheatgrass before we sampled and this may have been particularly likely behind houses where the owners had clearly manicured their alley property. However, these manicured alley properties were relatively uncommon (~5 out of 512 data points) and were not confounded with streetlight or pole presence.

Streetlights in Denver were traditionally sodium vapor lights, and these lights were present during the 2019 growing season. However, in late summer 2019 the city of Denver started to replace the sodium vapor lights for more efficient LED lights; whether this switch from sodium vapor to LED would lead to different effects in future years is unknown and should be tested, but during our study all plants grew under sodium vapor streetlights.

### 2.3 Statistical analysis

We had a total of 512 data points across 54 alleys. For each data point, we recorded whether the site lacked a pole (no pole, *n* = 239), had a pole without a streetlight (unlit pole, *n* = 192), or had a pole with a streetlight (lit pole, *n* = 81); the mean number of data points per alley was 9.5 and the range of data points was from 6 to 14 per alley. We first tested whether the *presence of plants* (present/absent) was affected by the type of *pole* (no pole, unlit pole, or lit pole). Then, using only the sites where plants were present, we tested whether the *presence of cheatgrass* (present/absent) was affected by the type of *pole* (no pole, *n* = 165; unlit pole, *n* = 164; or lit pole *n* = 77). Thus, our two response variables were *presence of plants* and *presence of cheatgrass*, our fixed effect was *pole*, and our random effect was *alley*. Estimates and means for our fixed effects are given with standard errors; our random effect is summarized by its variance, standard deviation, and marginal and conditional probabilities (*r* ^2^). We performed all analyses in R Studio 1.1453 (R Core Team, 2020) using the package lme4 (Bates et al., 2015) with glmer function with a binomial family.

## 3 RESULTS

**FIGURE 1:**
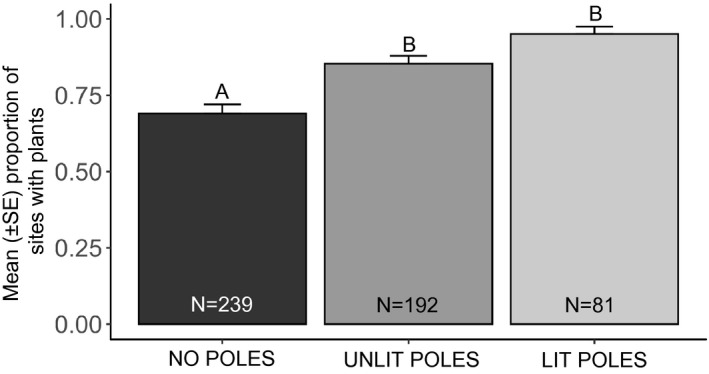
Mean proportion of surveyed sites with plants present depending on the presence/absence of (a) poles and (b) streetlights (August 2019; Denver, CO). Significant differences between means are indicated by letters above the bars, sample sizes are given for each treatment within its respective bar, and error bars show ±1 *SE*

We found that the presence of plants in an alley was dependent on the type of disturbance (*F*_2,466.2_ = 18.06, *p* < .0001). (Figure 1). Simultaneous pairwise comparisons using Tukey's HSD test indicated that the proportion of sites with plants was lower in sites without poles compared with sites with unlit poles (*Z* = −4.06, *p* = .001) or sites with lit poles (*Z* = −4.19, *p* = .001). The proportion of sites with plants did not differ between sites with unlit poles and lit poles (*Z* = −2.16, *p* = .08). The presence of plants also differed among alleys, which was our random effect (variance = 0.38 ± 0.62). The random effect of alley explained 23.8% of our model's variance compared with just the fixed effects (*r* ^2^ = .15).

**FIGURE 2:**
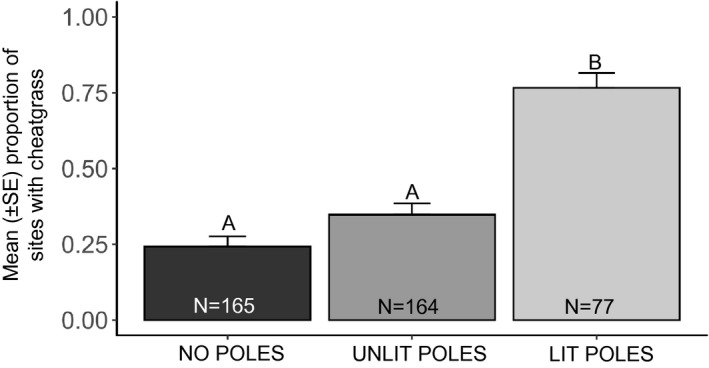
Mean proportion of surveyed sites with cheatgrass present depending on the presence/absence of (a) poles and (b) streetlights (August 2019; Denver, CO). Significant differences between means are indicated by letters above the bars, sample sizes are given for each treatment within its respective bar, and error bars show ±1 *SE*

For sites with plants, we found that the presence of cheatgrass in an alley was affected by light pollution (*F*_2,376.4_ = 37.88, *p* < .0001) (Figure 2). Specifically, sites with lit poles had the greatest presence of cheatgrass compared with either site with unlit poles (*Z* = −5.73, *p* < .0001) or sites without any poles (*Z* = −7.05, *p* < .0001). We found no statistical differences in the number of sites with cheatgrass at sites where poles were absent versus sites with unlit poles (*Z* = −2.15, *p* = .08). The presence of cheatgrass also differed among alleys, which was our random effect (variance = 0.20 ± 0.45). The random effect of alley explained 23.0% of our model's variance compared with just the fixed effects (*r* ^2^ = .18).

## 4 DISCUSSION

We found that artificial light positively affected the presence of plants generally and cheatgrass specifically in alleys. Only recently have researchers begun to investigate how artificial light at night may differentially affect native versus invasive plant species. Speißer et al. (2020) conducted the first experimental test to investigate how artificial light affects biomass production and competitive ability of naturalized alien plants. They found that when grown alone, widely naturalized alien plants did not respond to artificial light at night, but when grown in competition with less widely naturalized alien plants, the widely naturalized species produced significantly more biomass; these results suggest that widely naturalized alien plants may increase their competitive ability under artificial light. Interestingly, while the less widely naturalized alien plants were smaller overall, they responded positively to artificial light when grown alone and in competition with other species. Speißer et al. (2020) investigated plant species that are alien at their study site in Germany, but our research is the first to investigate how artificial light affects cheatgrass, which is an important invasive species in North America. Investigations into how artificial light may increase the performance of invasive plant species compared to native plants are a new area of inquiry and our research suggests that cheatgrass is one of these species that performs exceptionally well under artificial light conditions compared with other plant species.

For plants in general, we found that the presence of utility poles significantly increased the proportion of sites with plants, regardless of whether the poles were lit or unlit. Indeed, the proportion of sites with plants did not differ significantly between sites with unlit poles and sites with lit poles. For cheatgrass, we found that the presence of streetlights had a greater effect than the presence of poles. Cheatgrass was over 3× more common at sites with lit poles than at sites with unlit poles or no poles. Notably, the proportion of sites with cheatgrass did not differ significantly between sites with no poles and sites with unlit poles.

The presence of poles clearly had a facilitating effect on the presence of plants in general. In many alleys, there were gaps between a pole and the pavement, and these gaps created space around the poles for plants to establish. Generally, the soils in the alleys seemed compacted and it is possible that when utility crews service the poles, the resulting ground disturbance may facilitate seed dispersal into these areas with slightly less compacted soil. Road disturbance (e.g., breaking pavement) has previously been found to facilitate dispersal of invasive species (Dong et al., 2008), and our research suggests that disturbance associated with the installation of utility poles may facilitate plant growth. The variance among alleys as shown by our random effect suggests that alleys may have had other features (e.g., traffic, maintenance frequency) that also influenced our results.

Most research on cheatgrass invasion has occurred in rangelands or natural grasslands (e.g., Mack, 2011; Owens et al., 2013; Pawlak et al., 2015), but to our knowledge there are no studies of how cheatgrass invades urban ecosystems. Murphy et al. (In Review) found in a greenhouse experiment that cheatgrass had twice as much aboveground biomass when grown under streetlights compared with a control treatment, and indeed, none of the four native grasses tested grew larger under streetlights compared with their growth in the control treatment. Here, we show that streetlights may be facilitating cheatgrass growth outside the greenhouse in an urban environment. Notably, the effect of streetlights was greater than the effect of poles; at sites with poles, cheatgrass was found three times more often if the pole had a streetlight on it. Cheatgrass thus appears to thrive in the altered microhabitat created by streetlights.

As urbanization and artificial light increase across the western United States, our research suggests that the positive effect of streetlights on cheatgrass may be an important yet overlooked aspect of its invasion biology. We conducted this research as part of DU SciTech, which is a week‐long science camp for middle‐school girls designed to increase representation of women in STEM. At the end of camp, each participant proposed new questions and research directions that we could pursue in future years (Table 1). There are many unexplained topics to explore into the mechanisms that drive the pattern we found, including how our results may vary across neighborhoods with more urban or more suburban areas. The positive effect we found of streetlights on cheatgrass was before the city of Denver began to switch to more efficient LED lights, and whether these lights similarly affect cheatgrass is unknown.

**TABLE 1:** Future research questions that we devised during the University of Denver's (DU) SciTech STEM camp 2019

Questions
How much light pollution does there need to be to positively affect plants?
How far does the effect of one streetlight go in an alley?
How do the different kinds of streetlights (sodium vapor vs. LED) affect plants?
Does the color of the light matter to plant growth?
Is the number of streetlights the same across neighborhoods in Denver?
What happens if streetlights are not in alleys, but on streets?
Do streetlights affect snowmelt and soil moisture? Does this then affect the plants?
Do streetlights affect trees and other plants that live longer or just annual grasses?
Are the insects that feed on and pollinate the plants affected by lights?
Are other animals affected by the lights or cheatgrass?

Perhaps one of the biggest unknown questions now is whether light pollution can increase the invasiveness and competitive ability of an alien plant species. Artificial light at night is known to have many different effects on wild plants in natural or semi‐natural environments and these effects have been recently reviewed (Bennie et al., 2016). However, how artificial light may differentially affect native versus alien plants has only just started to be investigated (Speißer et al., 2020) and never in an urban environment such as where we conducted our study. Here, we showed that cheatgrass is more likely to be found at sites with streetlights, but future research is needed to investigate whether cheatgrass grown under artificial light has increased competitive ability compared with native species and/or other invasive species. We also need to determine the mechanism(s) that allow cheatgrass to outperform its plant neighbors. More broadly, however, more studies are needed to determine the role that artificial light at night may play in plant invasions and how widespread a phenomenon this may be.

## CONFLICT OF INTEREST

None declared.

## AUTHOR CONTRIBUTION

**Shannon M. Murphy:** Conceptualization (lead); Data curation (equal); Formal analysis (equal); Funding acquisition (lead); Investigation (equal); Methodology (equal); Project administration (lead); Supervision (lead); Visualization (equal); Writing‐original draft (lead); Writing‐review & editing (lead). **Dhaval K. Vyas:** Data curation (lead); Formal analysis (lead); Investigation (equal); Methodology (equal); Project administration (equal); Visualization (lead); Writing‐original draft (equal); Writing‐review & editing (equal). **Jennifer L. Hoffman:** Conceptualization (equal); Funding acquisition (lead); Investigation; Methodology; Project administration; Supervision (equal); Writing‐review & editing (equal). **Clara S. Jenck:** Conceptualization (equal); Investigation (equal); Methodology (equal); Project administration (equal); Writing‐review & editing (equal). **Brooke A. Washburn:** Conceptualization (equal); Investigation (equal); Methodology (equal); Project administration (equal); Writing‐review & editing (equal). **Kelsie E. Hunnicutt:** Conceptualization (equal); Investigation (equal); Methodology (equal); Project administration (equal); Writing‐review & editing (equal). **Angie Davidson:** Conceptualization (equal); Investigation (equal); Methodology (equal); Project administration (equal); Writing‐review & editing (equal). **Jasmine M. Andersen:** Conceptualization (equal); Investigation (equal); Methodology (equal); Project administration (equal); Writing‐review & editing (equal). **Rachel K. Bennet:** Conceptualization (equal); Investigation (equal); Methodology (equal); Project administration (equal); Writing‐review & editing (equal). **Amarin Gifford:** Conceptualization (equal); Investigation (equal); Methodology (equal); Project administration (equal); Writing‐review & editing (equal). **Mia Herrera:** Conceptualization (equal); Investigation (equal); Methodology (equal); Project administration (equal); Writing‐review & editing (equal). **Braelynn Lawler:** Conceptualization (equal); Investigation (equal); Methodology (equal); Project administration (equal); Writing‐review & editing (equal). **Sophie Lorman:** Conceptualization (equal); Investigation (equal); Methodology (equal); Project administration (equal); Writing‐review & editing (equal). **Vanessa Peacock:** Conceptualization (equal); Investigation (equal); Methodology (equal); Project administration (equal); Writing‐review & editing (equal). **Lyndi Walker:** Conceptualization (equal); Investigation (equal); Methodology (equal); Project administration (equal); Writing‐review & editing (equal). **Esme Watkins:** Conceptualization (equal); Investigation (equal); Methodology (equal); Project administration (equal); Writing‐review & editing (equal). **Lakyn Wilkinson:** Conceptualization (equal); Investigation (equal); Methodology (equal); Project administration (equal); Writing‐review & editing (equal). **Zariah Williams:** Conceptualization (equal); Investigation (equal); Methodology (equal); Project administration (equal); Writing‐review & editing (equal). **Robin M. Tinghitella:** Conceptualization (equal); Funding acquisition (lead); Investigation (equal); Methodology (equal); Project administration (equal); Supervision (lead); Writing‐review & editing (equal).

## Supporting information

Fig S1‐S2

## Data Availability

Data are archived at Zenodo: https://doi.org/10.5281/zenodo.4914145.
