# Supplementary material for: Streetlights positively affect the presence of an invasive grass species
Source: Ecol Evol. 2021 Jul 10;11(15):10320–6. doi: 10.1002/ece3.7835 (PMC8328438; doi:10.1002/ece3.7835)

**SUPPLEMENTARY INFORMATION**

**Figure S1.** Map of alley locations surrounding the University of Denver, located in Denver, Colorado, USA. The alleys that we sampled are all located within the red outlined areas to the east and west of the University of Denver.


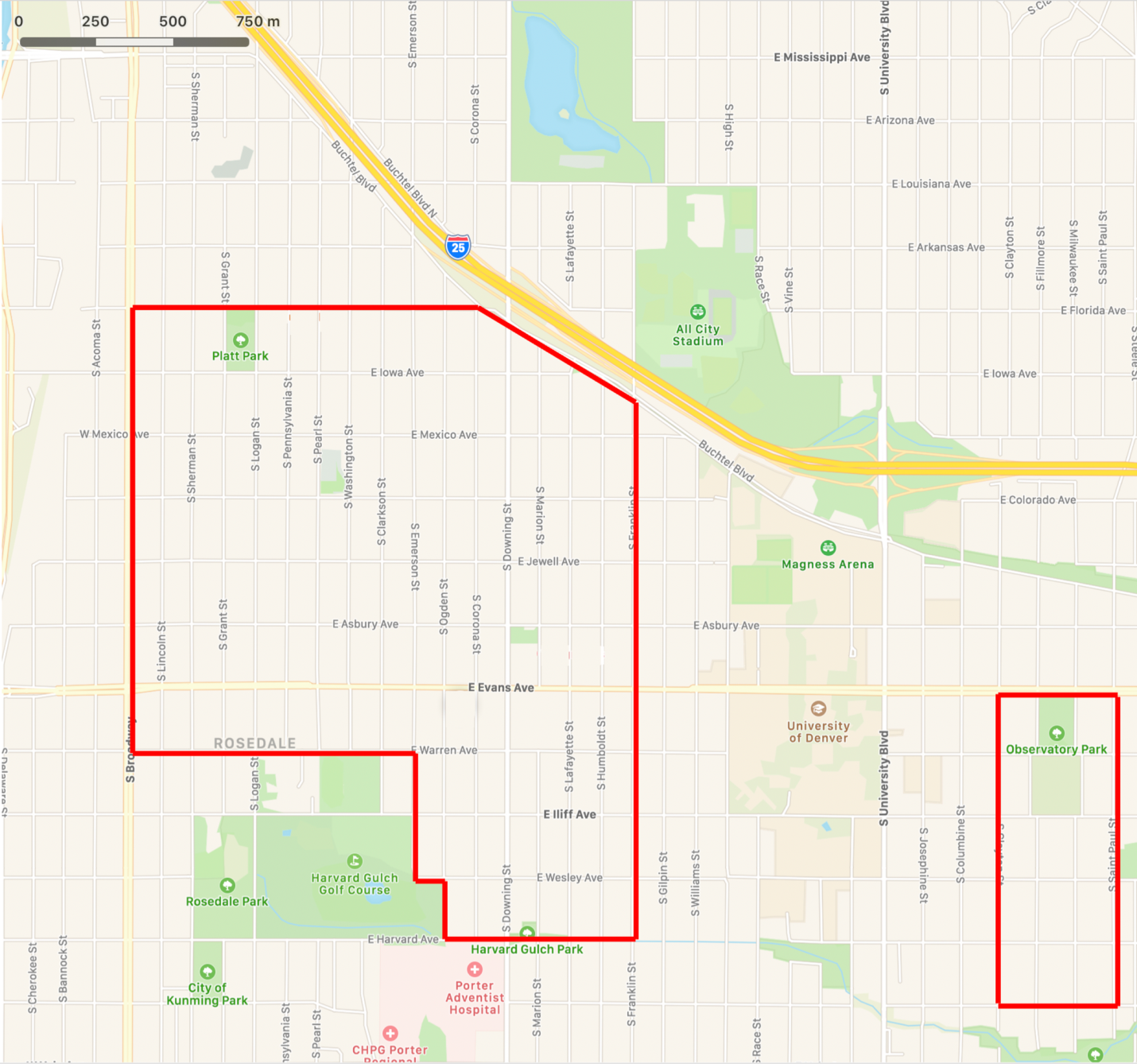


**N**

**Figure S2.** One of our alley sites located near the University of Denver with a streetlight mounted on the nearest pole (lit pole) and no streetlight mounted on the next farthest pole (unlit pole). Note that there are places for plants to grow along the alley, both near and away from poles.


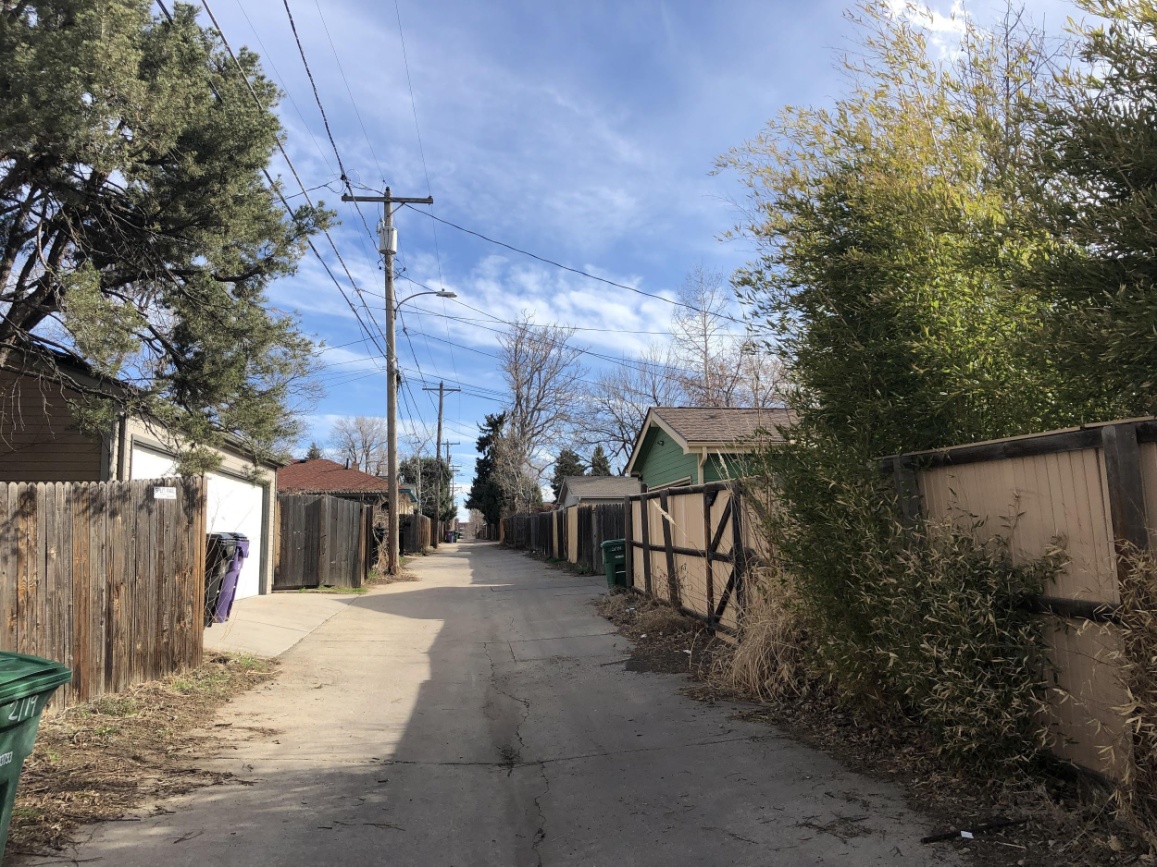

Supplement: Supplementary file 1 — Fig S1‐S2 [file ECE3-11-10320-s001.docx]
